# Polymerization of l-Tyrosine, l-Phenylalanine, and 2-Phenylethylamine as a Versatile Method of Surface Modification for Implantable Medical Devices

**DOI:** 10.1021/acsomega.2c05289

**Published:** 2022-10-20

**Authors:** Kamil Kopeć, Agata Ryżko, Roman Major, Hanna Plutecka, Justyna Wiȩcek, Grzegorz Pikus, Jakub W. Trzciński, Adrianna Kalinowska, Tomasz Ciach

**Affiliations:** † Faculty of Chemical and Process Engineering, Biomedical Engineering Laboratory, Warsaw University of Technology, Waryńskiego 1, Warsaw 00-645, Poland; ¶ Department of Cytology, Faculty of Biology, University of Warsaw, Miecznikowa 1, Warsaw 02-089, Poland; ‡ Institute of Metallurgy and Materials Science, Polish Academy of Sciences, Reymonta 25, Cracow 30-059, Poland; § Department of Medicine, Jagiellonian University Medical College, Skawińska 8, Cracow 31-066, Poland; ∥ School of Chemistry, University of Bristol, Cantock’s Cl, Bristol BS8 1TS, United Kingdom; ⊥ Centre for Advanced Materials and Technologies CEZAMAT, Warsaw University of Technology, Poleczki 19, Warsaw 02-822, Poland

## Abstract

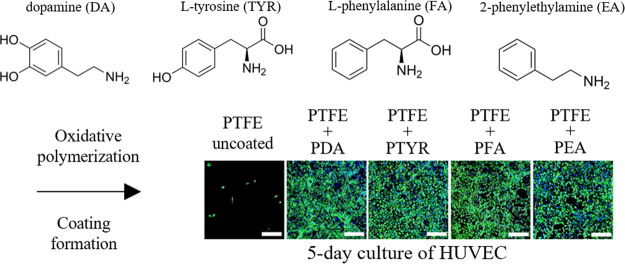

Surface properties are crucial for medical device and implant research and applications. We present novel polycatecholamine coatings obtained by oxidative polymerization of l-tyrosine, l-phenylalanine, and 2-phenylethylamine based on mussel glue-inspired chemistry. We optimized the reaction parameters and examined the properties of coatings compared to the ones obtained from polydopamine. We produced polycatecholamine coatings on various materials used to manufacture implantable medical devices, such as polyurethane, but also hard-to-coat polydimethylsiloxane, polytetrafluoroethylene, and stainless steel. The coating process results in significant hydrophilization of the material’s surface, reducing the water contact angle by about 50 to 80% for polytetrafluoroethylene and polyurethane, respectively. We showed that the thickness, roughness, and stability of the polycatecholamine coatings depend on the chemical structure of the oxidized phenylamine. In vitro experiments showed prominent hemocompatibility of our coatings and significant improvement of the adhesion and proliferation of human umbilical vein endothelial cells. The full confluence on the surface of coated polytetrafluoroethylene was achieved after 5 days of cell culture for all tested polycatecholamines, and it was maintained after 14 days. Hence, the use of polycatecholamine coatings can be a simple and versatile method of surface modification of medical devices intended for contact with blood or used in tissue engineering.

## 1 Introduction

The material/environment interface is essential for its interactions with biological systems. This is especially important for implants and other medical devices. Hence, the need for versatile and easy to produce coatings grows and stimulates research. Polydopamine (PDA) is one of the most interesting biomimetic polymers used to coat medical devices due to its capability to form a polymer layer on various surfaces by self-polymerization. The discovery of this phenomenon was inspired by research on adhesive proteins secreted by mussels.^1^ The PDA is a biocompatible polymer that creates a stable hydrophilic coating on virtually any material, becoming a base for further water-based surface modifications employing thiol or amine groups. These properties make PDA widely used in cell adhesion and patterning, bone and tissue engineering applications, biosensors, biomolecule immobilization, enhancement of biocompatibility, and antimicrobial applications.^2,3^ However, the synthesis of other polycatecholamine coatings as an alternative to PDA has not yet been studied.

Our approach was to adopt a dopamine oxidative polymerization method to synthesize analogous polycatecholamine coatings obtained from substrates other than expensive dopamine. We selected phenylamines that contain an aromatic ring and an amino group connected by a short aliphatic chain, analogous to the structure of dopamine. We assumed that using a strong oxidant would lead to introduction of the hydroxyl groups into the aromatic ring of phenylamines to form a catechol moiety. The products of phenylamine oxidation, containing catechol groups, should undergo polymerization and coating formation, analogous to PDA. The use of dopamine analogs enhances the ability to introduce new chemical groups like carboxylic acids, amines, or aromatic hydrocarbons and significantly reduces surface modification costs.

The described study proposes a new method of synthesizing polycatecholamine coatings from various phenylamines: dopamine, l-tyrosine, l-phenylalanine, and 2-phenylethylamine. This work aims to find the most effective synthesis method and compare the chemical and physical properties of obtained coatings. We produced polycatecholamine coatings on the surface of polydimethylsiloxane, polytetrafluoroethylene, polyurethane, and stainless steel, which are the materials used to manufacture implantable medical devices, such as urological and cardiological catheters, coronary stents, intra-articular implants, bone scaffolds, and vascular prostheses.^4^ Hemocompatibility evaluation and in vitro determination of cytocompatibility were performed to test the utility of the new coatings in biomedical applications. Moreover, the potential mechanism of phenylamine polymerization and coating formation has also been discussed.

## 2 Experimental Section

### 2.1 Materials and Chemicals

Dopamine hydrochloride (99%) was purchased from Alfa Aesar (Kandel, Germany). l-Tyrosine (≥99%) was purchased from Carl Roth GmbH (Karlsruhe, Germany). l-Phenylalanine (≥98.5%), 2-phenylethylamine (99%), sodium periodate (≥99.8%), sodium persulfate (≥98%), hydrogen peroxide 30% (w/w) in H_2_O, iron(II) chloride (98%), sulfuric acid (95–97%), acetic acid (≥99%), sodium acetate (≥99%), acetone (≥99.5%), phosphate-buffered saline tablets, sodium dodecyl sulfate (≥99.8%, SDS), adenosine diphosphate, F-12K Complete Medium, antibiotic antimycotic solution, paraformaldehyde (reagent grade), and Triton X-100 were purchased from Sigma-Aldrich Chemie GmbH (Steinheim, Germany). Alexa Fluor 488 Phalloidin, DAPI, and DRAQ5 staining solution dyes were purchased from Thermo Fisher Scientific (Waltham, USA). Antibodies PerCP anti-CD61, FITC anti-PAC-1, and PE-anti-CD62P were purchased from BD Biosciences (New Jersey, USA). The cell line of human umbilical vein endothelial cells (HUVECs) was obtained from ATCC (Manassas, USA). Silicon wafers (thickness = 625 ± 25 μm, 1–10 Ωcm, one-side polished, p-type) were purchased from MicroChemicals GmbH (Ulm, Germany). Polymethyl methacrylate (PMMA) spectrophotometric cuvettes were purchased from VWR International (Gdansk, Poland). Quartz spectrophotometric cuvettes (21QS10) were purchased from Biosens (Warsaw, Poland). SYLGARD 184 polydimethylsiloxane (PDMS) was purchased from Dow Corning (Midland, USA). A polytetrafluoroethylene (PTFE) fluoroplast-4 tape was purchased from HaloPolymer (Moscow, Russia). ChronoFlex C75D polyurethane (PU, medical grade) was purchased from AdvanSource Biomaterials Corporation (Wilmington, USA). Stainless steel 316 L (SS) discs of a diameter of 14 mm were purchased from STOMILEX (Piastów, Poland).

UV-Vis spectra and the absorbance at 400 nm of polycatecholamine coatings created on the inner walls of the quartz or PMMA spectrophotometric cuvettes were collected using a UV-Vis spectrophotometer Helios Gamma 9423 UVG 1702E (Thermo Fischer Scientific, Horsham, United Kingdom). FTIR-attenuated total reflection (ATR) spectra of the polycatecholamine powders were collected using a FTIR spectrometer Nicolet 6700 (Thermo Scientific, Schwerte, Germany). The thickness, roughness, and topography of the polycatecholamine coatings were investigated using an atomic force microscope diMultiMode V with Nanoscope V Controller (Veeco, Plainview, USA) with an ACSTA-50 probe (AppNano, Mountain View, USA). The wettability of the tested materials was studied using a goniometer DSA100 (Krüss GmbH, Hamburg, Germany). The topography of the polycatecholamine coatings was imaged using a FEI Phenom scanning electron microscope (Phenom-World, Eindhoven, the Netherlands). Samples before imaging by SEM were sputtered with gold using a sputter K550X Emitech (Quorum Technologies, Laughton, UK). A cone-and-plate(let) analyzer (CPA, Impact-R, DiaMed AG, Switzerland) and a BD FACSCanto II flow cytometer (BD Biosciences, New Jersey, USA) were used in the hemocompatibility evaluation of polycatecholamine coatings. Cells cultured on materials with polycatecholamine coatings were visualized using a confocal laser scanning microscope (LSM 880, Zeiss, Sheung Kehen, Germany).

### 2.2 Synthesis of Polycatecholamine Coatings

#### 2.2.1 Preparation of Surfaces of Coated Materials

All materials (excluding spectrophotometric cuvettes) before producing polycatecholamine coatings on their surface were rinsed in deionized water, acetone, and deionized water. After that, the materials were immersed in the piranha solution (sulfuric acid and 30% hydrogen peroxide solution in a 1:1 volume ratio) for 10 s, rinsed thoroughly in deionized water, and used without drying. The coatings on the inner walls of the spectrophotometric cuvettes were produced without surface pretreatment.

#### 2.2.2 Synthesis of Polydopamine (PDA V-I and PDA V-II) Coatings

The most effective method of PDA coating synthesis was studied by selecting the dopamine polymerization process parameters: type of oxidizing agent, pH, dopamine concentration, the molar ratio of dopamine to the oxidizing agent, and temperature. Dopamine polymerization was carried out by filling the PMMA spectrophotometric cuvettes with the coating solution. The coating solution was removed from the cuvette at the tested time points. The cuvette was rinsed with distilled water and dried at 60 °C for 10 min. Then, the cuvette was filled with distilled water, and the absorbance of the PDA film created on the inner walls was measured at 400 nm using the UV-Vis spectrophotometer against distilled water. Three samples were measured at each time point (*n* = 3). In the first step, the influence of the dopamine-oxidizing agent was investigated. Atmospheric oxygen, persulfate, hydrogen peroxide, periodate at pH 8.5, and periodate at pH 5.0 were tested. Each reaction was performed at 25 °C in a 2.0 mg/mL dopamine solution in which the molar ratio of dopamine to oxidant was 2:1. The process conditions were determined sequentially for the selected oxidizing agent in the following steps.

The parameters of the PDA coating synthesis selected as described above were used for further studies. The coatings on the surface of flat materials (silicon, PDMS, PTFE, PU, SS) were synthesized by immersing the substrate in a coating solution containing 2.0 mg/mL dopamine hydrochloride with the addition of sodium periodate in a dopamine:periodate molar ratio of 2:1 in 50 mM acetate buffer at pH 5.5. The process was carried out with magnetic stirring at 300 rpm for 1 h at 25 °C. The coated material was washed successively with distilled water, 0.1% SDS solution, and distilled water again. Then, the samples were dried at 60 °C for 10 min. The PDA-coated samples in this process were further designated as PDA V-II.

PDA coating synthesized according to the most common method, employing atmospheric oxygen as an oxidizing agent, was also used to compare with the polycatecholamine coatings developed in this work. The coatings were synthesized by immersing the substrate in a coating solution containing 2.0 mg/mL dopamine hydrochloride in 10 mM Tris–HCl buffer at pH 8.5. The process was carried out with magnetic stirring at 300 rpm for 24 h at 25 °C. The coated substrate was washed successively with distilled water, 0.1% SDS solution, and distilled water again. Then, the samples were dried at 60 °C for 10 min. The PDA-coated samples in this process were further designated as PDA V-I.

#### 2.2.3 Synthesis of Polytyrosine (PTYR) Coatings

The synthesis of PTYR coatings was performed using the Fenton reaction, where the oxidation of l-tyrosine takes place with the participation of the hydroxyl radical. Preliminary studies have shown that a supersaturated l-tyrosine solution is required to produce PTYR and the acidic pH is preferred. The selection of the best process conditions for the synthesis of PTYR coatings was made using the Box–Behnken plan using STATISTICA 12.5 software (StatSoft Inc., Tulsa, USA). Fixed parameters l-tyrosine concentration 0.8 mg/mL and temperature 25 °C and variable parameters pH 2.0, 4.0, and 6.0; FeCl_2_ concentrations 0.1, 0.5, and 0.9 mM; and H_2_O_2_:FeCl_2_ molar ratios 5:1, 25:1, and 45:1 were selected. The absorbance at 400 nm of the PTYR film formed on the inner walls of the PMMA spectrophotometric cuvette after 1 h of the reaction at 25 °C was the output parameter measured using the UV-Vis spectrophotometer against distilled water. Three samples were measured at each time point (*n* = 3). For selected process parameters, the influence of temperature on the PTYR coating formation rate was examined similarly.

The parameters of the PTYR coating synthesis selected as described above were used for further studies. The coatings on the surface of flat materials (silicon, PDMS, PTFE, PU, SS) were synthesized by immersing the substrate in a 0.8 mg/mL l-tyrosine solution at pH 4.0 (pH value adjusted with 1 M HCl). Then, FeCl_2_ and H_2_O_2_ were added to obtain a final coating solution with a FeCl_2_ concentration of 0.6 mM and a H_2_O_2_:FeCl_2_ molar ratio of 25:1. The process was carried out with magnetic stirring at 300 rpm for 24 h at 25 °C. The coated material was washed with distilled water, 0.1% SDS solution, and distilled water again. Then, the samples were dried at 60 °C for 10 min.

#### 2.2.4 Synthesis of Polyphenylalanine (PFA) and Polyphenylethylamine (PEA) Coatings

The PFA and PEA coatings on the surface of flat materials (silicon, PDMS, PTFE, PU, SS) were synthesized by immersing the substrate in a 4.0 mg/mL solution of l-phenylalanine or 2-phenylethylamine at pH 4.0 (pH value adjusted with 1 M HCl). Then, FeCl_2_ and H_2_O_2_ were added to obtain a final coating solution with a FeCl_2_ concentration of 1.5 mM and a H_2_O_2_:FeCl_2_ molar ratio of 25:1. The process was carried out with magnetic stirring at 300 rpm for 24 h at 25 °C. The coated material was washed successively with distilled water, 0.1% SDS solution, and distilled water again. Then, the samples were dried at 60 °C for 10 min.

### 2.3 Characterization of Polycatecholamine Coatings

#### 2.3.1 UV-Vis Spectroscopy

Polycatecholamine films were formed inside the quartz spectrophotometric cuvettes using coating solutions and synthesis parameters described in sections 2.2.2–2.2.4. After removing the coating solutions, the cuvettes were washed with distilled water three times, dried at 60 °C for 10 min, and the absorbance of the polycatecholamine films was measured at room temperature using the UV-Vis spectrophotometer against distilled water.

#### 2.3.2 Attenuated Total Reflectance-Fourier Transform Infrared (ATR-FTIR) Spectroscopy

The chemical structure comparison was made by ATR-FTIR analysis of the powders of the synthesized polycatecholamines. The synthesis of individual polycatecholamines was performed as described in sections 2.2.2–2.2.4. but without immersing any substrate. After the specified time (24 h for PDA V-I, PTYR, PFA, and PEA and 1 h for PDA V-II), the precipitate of polycatecholamines was separated by centrifugation at 4500 rpm for 25 min. The powders were purified by washing with distilled water and centrifugation (4500 rpm for 25 min), repeated five times. Polycatecholamine powders were analyzed using the FTIR spectrometer. The spectra were detected in ATR mode and analyzed with the OMNIC 8.3 software. Spectra were recorded for at least four different samples for each polycatecholamine. One characteristic spectrum for each tested material was selected for presentation.

#### 2.3.3 Atomic Force Microscopy (AFM)

The thickness, roughness, and topography of the polycatecholamine coatings were investigated using AFM. The coatings were prepared on a silicon surface as described in sections 2.2.2–2.2.4. For thickness measurements, a portion of the coating was removed from the sample with a steel razor blade and the difference in height between the coated and uncoated areas of the sample was measured. Five edge areas were tested for each coating (*n* = 5). Roughness and topography were analyzed for at least 25 μm^2^ scan areas. Roughness parameters (Ra and R_Z_) were determined for five scan areas for each coating (*n* = 5). One characteristic image for each tested coating was selected for surface topography presentation.

#### 2.3.4 Wettability Measurements

The ability of polycatecholamine coatings to surface hydrophilization was tested for four different materials: PDMS, PTFE, PU, and SS. The coatings were prepared as described in sections 2.2.2–2.2.4. The wetting properties of the tested materials were studied using the goniometer and the sessile drop method. Five microliters of distilled water droplets was dispensed on the tested materials, and the water contact angle values were measured using Advance 1.4.1.2 software. For each material and coating, three independent samples were tested with three water droplets for each sample (*n* = 9).

#### 2.3.5 Scanning Electron Microscopy (SEM)

The topography of the polycatecholamine coatings was investigated using SEM. The coatings were prepared on the PU surface as described in sections 2.2.2–2.2.4. Samples of coated PU were sputtered with about a 10 nm layer of gold and imaged using the scanning electron microscope in topographic mode. The images were recorded for at least four different places for each polycatecholamine coating. One characteristic image for each tested material was selected for presentation.

#### 2.3.6 Stability Measurements

Polycatecholamine films were formed inside PMMA spectrophotometric cuvettes using coating solutions and synthesis parameters described in sections 2.2.2–2.2.4. Then, cuvettes were filled with 0.01 M phosphate-buffered saline (PBS) at pH 7.4 and incubated at 37 °C. After 1, 2, 3, and 4 weeks, the cuvettes were emptied, rinsed three times with distilled water, and filled with distilled water, and the absorbance at 400 nm of the polycatecholamine films remaining on the inner walls was measured. The same cuvettes were then refilled with PBS and incubated until the next time point. The absorbance was measured for five independent samples (*n* = 5) for each polycatecholamine coating using the UV-Vis spectrophotometer against distilled water.

### 2.4 Hemocompatibility Evaluation of Polycatecholamine Coatings

The CPA was used in the present study. Following the instruction given by the producer, 130 μL blood volume was used for the experiment. The blood was mixed for 60 s on the rotational wheel to prevent sedimentation. The reference material stated polystyrene (PS) surface, which was the original disposable insert well of the Impact-R kit. Polycatecholamine coatings were produced on the PDMS surface as described in sections 2.2.2–2.2.4. All tested materials were cut out as discs of 14.4 mm diameter and 2 mm thickness, which fitted the insert well. The details of the method are described elsewhere.^5^ The shear test was applied at a shear rate of 1800 1/s for 300 s, using a disposable PTFE conical rotor. Following the shear test, the rotor was carefully removed, and blood was immediately sampled from the well to the test tubes for flow cytometry staining. From the remaining blood (80 μL), plasma was separated by centrifugation at 4000 g for 6 min and stored frozen at −80 °C for further analysis of thrombotic activity. The cone-and-plate test was developed to study the effect of shear stresses on cardiovascular tissue. Initially, it was designed for laminar flows as a system of two parallel plates to obtain a uniform shear stress distribution. Later modifications were done to obtain oscillating shear stresses. The cone-and-plate is another system of this test designed to obtain a uniform distribution of shear stresses and was initially used to measure the viscosity of the liquid. Currently, pulsed and oscillating shear stresses are generated in this system due to high shear rates. The main flow will generate the primary stress oriented in the tangential direction. An evaluation of platelet activation was carried out with the use of the flow cytometer. After the experiments, blood samples were stained with the following antibodies: PerCP anti-CD61, FITC anti-PAC-1, and PE-anti-CD62P. Positive events for both CD61 and CD62P (Selectin-P) or PAC-1 were assumed as activated platelets. In addition to the dynamic test, the following two blood samples stored under static conditions were also tested: unactivated (baseline (bas)) and activated by the addition of adenosine diphosphate (ADP) with the final concentration of 20 μM. Tests of each surface were repeated at least three times (*n* = 3).

### 2.5 Cell Culture on Materials with Polycatecholamine Coatings

#### 2.5.1 Culture of HUVECs

HUVECs were cultured in 250 mL 75 cm^2^ culture bottles (Falcon) in F-12K medium supplemented with 10% fetal bovine serum (FBS), 0.1 mg/mL heparin, 30 μg/mL endothelial cell growth supplement, and 1 μg/mL hydrocortisone at a humidified atmosphere with 5% CO_2_ at 37 °C (standard condition). The medium was changed every 2 or 3 days. HUVECs of passages 3–4 were used in all experiments. At 70–80% confluence, cells were detached from the bottom of the bottle by incubation in a non-enzymatic cell dissociation solution in standard conditions (37 °C, 5% CO_2_) for 15 min. After that, the HUVEC-specific medium (supplemented F-12K) was added to the bottle. The number of cells was counted using a Thoma chamber. Cells prepared in this way were used to start a HUVEC cell culture on the tested materials.

#### 2.5.2 Cell Seeding on Tested Materials

The polycatecholamine coatings for cell culturing were prepared on the surface of PTFE and PU as described in sections 2.2.2–2.2.4. Before biological tests, all materials were sterilized. Discs of 1 cm diameter were cut from the materials and placed in 48-well plates with 250 μL of antibiotic solution (0.25 μg/mL amphotericin, 100 U penicillin, 100 μg/mL streptomycin) dissolved in PBS without ions for 24 h at −4 °C in the dark. The insert made of the wider 0.5 cm long part of the 1 mL tips was placed into each well to keep the materials immersed in the solution throughout incubation. After 24 h, the materials were washed three times with PBS and pre-incubated in 500 μL of supplemented F-12K medium under standard conditions (37 °C, 5% CO_2_) for 2–3 h. Afterward, the medium was removed, and HUVECs (5 × 10^4^ cell/sample) were seeded on each material and incubated in supplemented F-12K medium at 37 °C in a humidified atmosphere with 5% CO_2_ for 1, 3, 5, and 14 days. The culture media were changed every second day. The inserts were removed from the wells only when removing or adding solutions to the wells. These cultures were used to study morphology and cell proliferation on the examined surfaces.

#### 2.5.3 Cell Staining and Visualization

After the desired incubation period, cells were washed three times with PBS without ion heated to 37 °C. Then, they were fixed by adding 4% (w/v) paraformaldehyde for 24 h at 4 °C and washed four times with PBS. Then, cells were permeabilized with 0.2% (v/v) Triton X-100 in PBS for approximately 8 min and washed three times with PBS. The samples were then incubated in 2.5% Alexa Fluor 488 Phalloidin dye solution in PBS for 1 h and then washed three times with PBS in the dark. Plates with discs were placed on a shaker for 5 min during all washes. To visualize nuclei, cells were incubated in the dark for 6 min in a DRAQ5 solution diluted 1:1000 with PBS. Finally, discs were washed three times with PBS, then pressed and fixed between the glass slides using a mounting medium with DAPI. All the steps were carried out at room temperature if not specified otherwise. At each stage, the discs were held by inserts at the bottom of the wells. Stained cells were visualized using the confocal laser scanning microscope at 10× magnification.

### 2.6 Statistical Analysis

The normality of distribution of wettability and roughness results was tested using the Kolmogorov–Smirnov test (*p* < 0.05). The difference between the mean values of measured parameters was tested in one-way ANOVA (*p* < 0.05) with post hoc Tukey’s test for multiple comparisons. Statistical analysis was performed using OriginPro 8 software (OriginLab Corporation, Northampton, USA).

## 3 Results and Discussion

The most commonly used method of PDA synthesis is the oxidation of dopamine solution in an alkaline environment using atmospheric oxygen as an oxidizing agent. This process only occurs when the reaction pH is higher than 8.^3,6^ However, it was proven that dopamine polymerization could be more efficient when oxidants other than atmospheric oxygen are used, such as ammonium persulfate,^7^ sodium periodate,^8,9^ and hydrogen peroxide,^10^ and in the presence of metallic catalysts (including Fenton reaction),^11−15^ frequently in acidic conditions.

**Figure 1:**
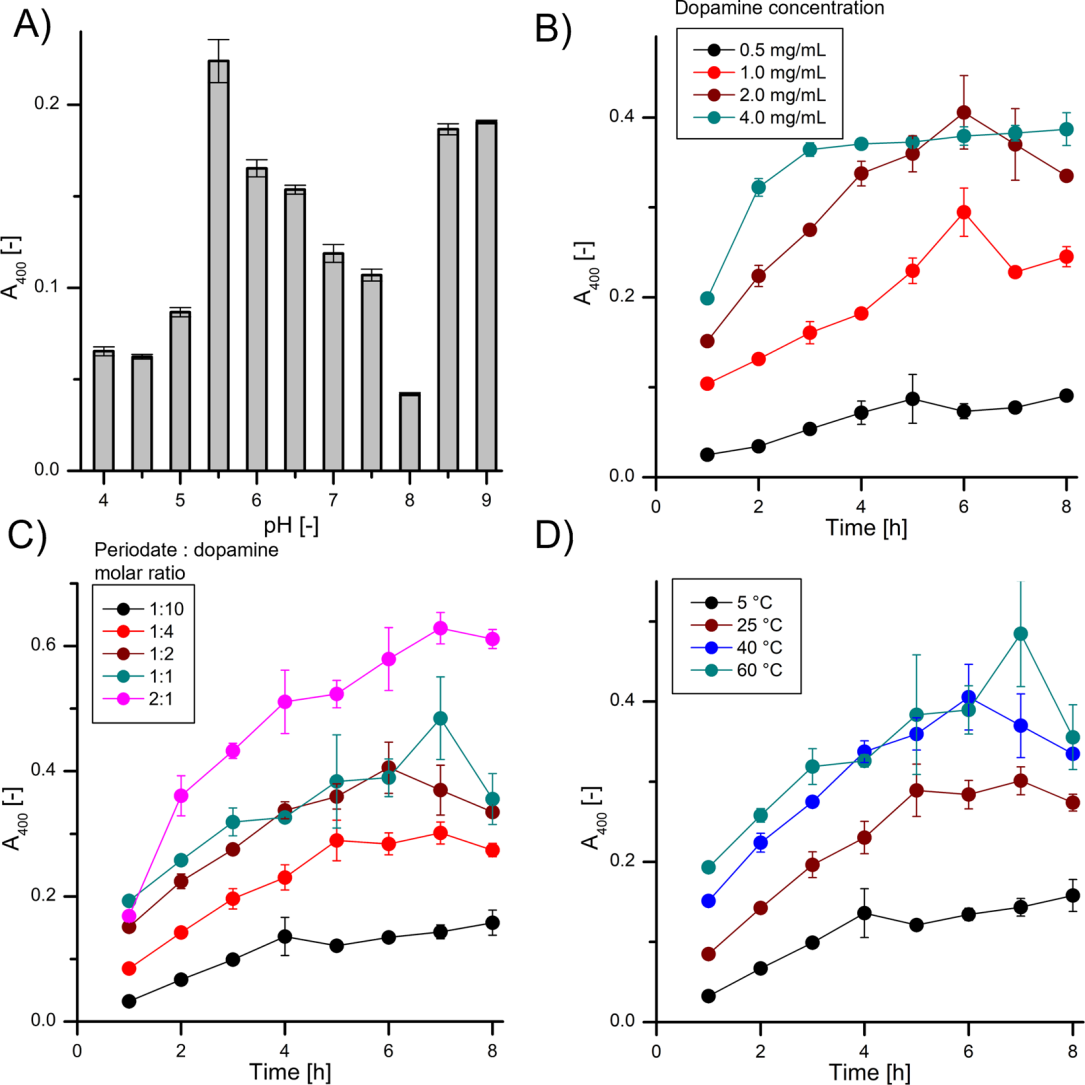
Influence of process parameters on the rate of periodate-oxidized PDA film formation on the inner walls of PMMA spectrophotometric cuvettes. (A) PDA absorbance at 400 nm as a function of pH for a 2-h process of PDA film formation; PDA absorbance at 400 nm as a function of time for various: (B) dopamine concentrations; (C) periodate:dopamine molar ratios; (D) temperatures.

Our preliminary study focused on the rate of PDA film growth using different oxidizing agents: atmospheric oxygen, persulfate, perhydrol, and sodium periodate. Among them, the highest and most stable PDA formation rate was found for sodium periodate at pH 5.0 (Figure S1). For this oxidant, we investigated the impact of the process parameters on the formation rate of the PDA films. As shown in Figure 1A, there is the maximum of PDA film absorbance at pH 5.5. This means that the maximum rate of dopamine oxidation with periodate occurs at pH 5.5. When the pH is neutral and slightly basic, the rate of dopamine oxidation with periodate is significantly reduced. The PDA film begins to regrow when the pH is higher than 8.0 as oxidation with atmospheric oxygen also begins. However, the oxidation of dopamine with periodate in an acidic environment is faster than oxidation with atmospheric oxygen in an alkaline environment, especially in the first hours of the process. We selected pH 5.5 for further experiments because of the highest absorbance value and the single mechanism of dopamine oxidation only by periodate. The rate of PDA film formation increases with increasing dopamine concentration (Figure 1B). Since concentrations of 2.0 and 4.0 mg/mL have the same absorbance at the time of 5 h, with a negatable difference at the time of 8 h, we decided to select the lower value making the reaction more controllable and less cost consuming. Moreover, the selected concentration is the most used in the literature for the atmospheric oxygen oxidation method, which makes our results more comparable to other studies.^3,6^ In the next step, we showed that an increase in the amount of periodate to dopamine causes an increase in the rate of PDA film formation (Figure 1C). Using a periodate molar ratio higher than 1:2 does not significantly increase the amount of PDA film in the first hour of the process, so this value was selected. If the goal is to obtain a very thick PDA coating, using a 4:1 molar ratio of periodate to dopamine and running the process for 7 h would be beneficial. However, using less oxidant is preferred for future biomedical applications. As shown in Figure 1D, the PDA film-forming process occurs even at the lowest tested temperature of 5 °C. Consequently, the increase in the reaction temperature up to 60 °C increases the rate of the process. Since the difference between 25 and 60 °C has a low effect on this reaction, we decided to conduct experiments at 25 °C as it provides the desired thickness of the coatings in the process that is easier to control and more cost-effective.

**Figure 2:**
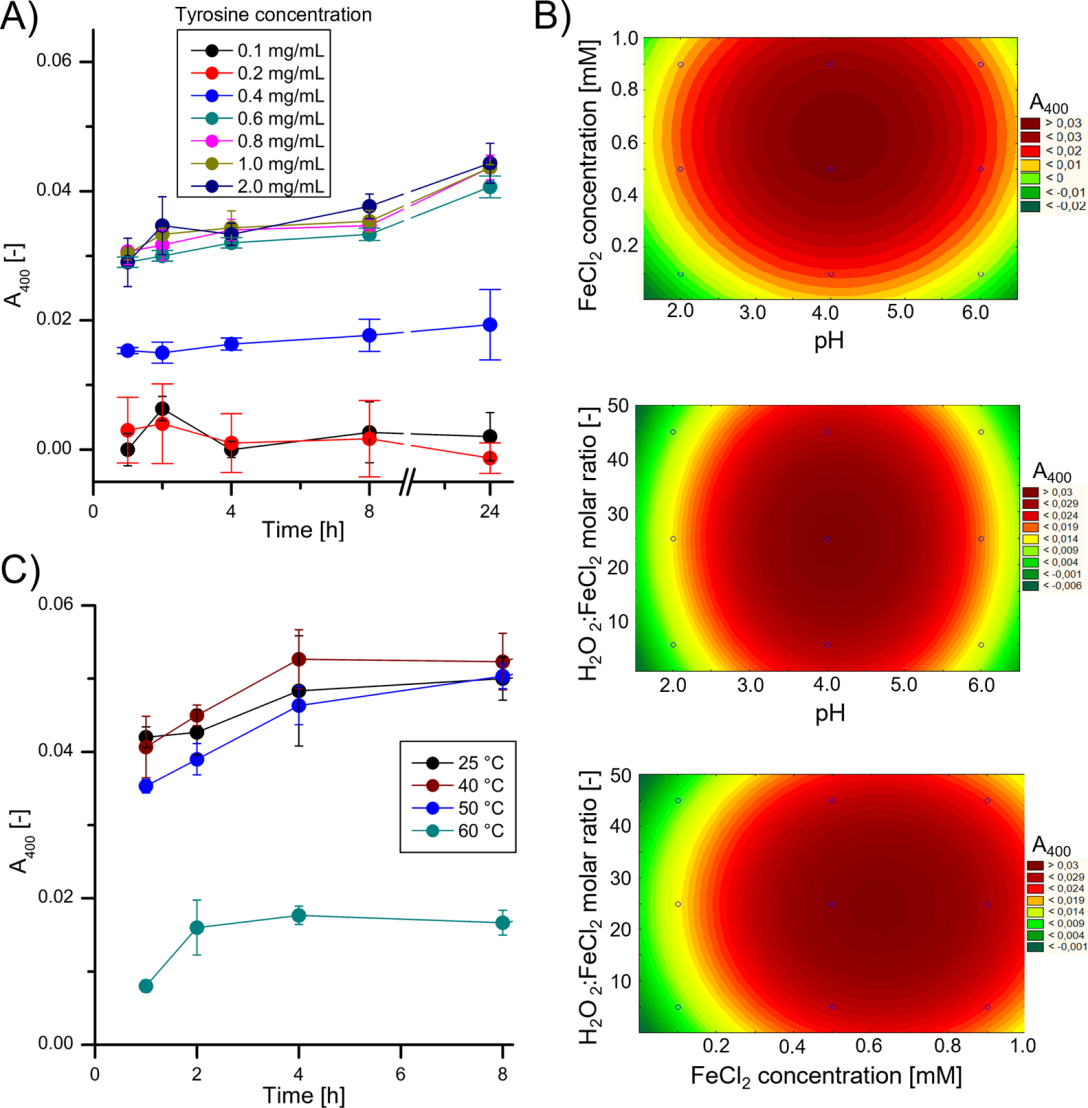
Influence of process parameters on the rate of Fenton-oxidized PTYR film formation on the inner walls of PMMA spectrophotometric cuvettes. (A) Influence of l-tyrosine concentration; (B) The 2D plots showing the combined effect of (from the top) FeCl_2_ concentration and pH, H_2_O_2_:FeCl_2_ molar ratio and pH, H_2_O_2_:FeCl_2_ molar ratio, and FeCl_2_ concentration, on PTYR absorbance at 400 nm for a 2-h process of PTYR film formation; (C) PTYR absorbance at 400 nm as a function of time for various temperatures.

The use of atmospheric oxygen, persulfate, hydrogen peroxide, and periodate did not result in polymerization and coating formation in the case of selected phenylamines: l-tyrosine, l-phenylalanine, and 2-phenylethylamine. Because of lower chemical reactivity, those phenylamines can form a polymeric film when a radical reaction, like Fenton,^16^ is applied. Therefore, we used this iron(II)-driven reaction for l-tyrosine polymeric coating synthesis. We have screened reaction parameters pH, FeCl_2_ concentration, H_2_O_2_:FeCl_2_ molar ratio, time, and the reaction temperature, in order to select the best conditions for the PTYR coating production. In the first step, we examined the effect of the l-tyrosine concentration. As shown in Figure 2A, the PTYR film is practically not formed when the l-tyrosine concentration is 0.2 mg/mL or less. Our study results show that the PTYR film is formed effectively at the concentration of 0.4 mg/mL, in which the l-tyrosine solution is close to supersaturation. When the l-tyrosine solution is supersaturated, the amount of PTYR film produced increases significantly. This increase is stopped when the l-tyrosine concentration reaches 0.6 mg/mL. This is because only the dissolved substrate is involved in the reaction. When l-tyrosine in the solution is oxidized with hydroxyl radicals, and successfully polymerized, a new portion of this amino acid is dissolved, maintaining its supersaturation. Therefore, for further research, we used the concentration of 0.8 mg/mL to ensure that an adequate supply of l-tyrosine in the suspension is provided and to avoid the l-tyrosine concentration which will be the limiting factor in selecting other process parameters. The selection of concentrations of other reagents and pH for the synthesis of PTYR coatings was made using the Box–Behnken plan.^17^ The plan of experiments and results are presented in Table S1. As shown in Figure 2B, the visualization of the experimental results indicates that the FeCl_2_ concentration of 0.6 mM, the H_2_O_2_:FeCl_2_ molar ratio of 25:1, and pH 4.0 are the most effective process parameters to produce PTYR films.

The temperature variation from 25 to 50 °C has no significant influence on the efficiency of the PTYR film formation (Figure 2C). However, the efficiency of the process decreases significantly when the temperature reaches 60 °C. This may be due to the mechanism of the polycatecholamine coating formation. Other authors assume that catecholamine is oxidized to several different products in the first step of coating formation.^9,18^ The oxidation products react then to water-soluble oligomers that adhere greatly to many surfaces. After that, other oxidation products are attached to the oligomers on the material’s surface, creating a final insoluble coating.^9,18^ At the highest tested temperature, the Fenton reaction is very fast, possibly causing the oligomers to be present in the solution for a very short time. Only a few oligomers adhere to the surface before being further polymerized in the solution. Also, the remaining products of catecholamine oxidation can be used mainly for polymerization in the solution volume, forming visible polycatecholamine aggregates. Increasing the temperature further enhances the rate of oxidation of catecholamines, possibly leading to the formation of thinner films on the surface of the coated materials and the production of more polycatecholamine aggregates in the solution.

Preliminary studies have shown that the polymerization of l-phenylalanine and 2-phenylethylamine and the production of PFA and PEA coatings by the Fenton reaction are less efficient than those processes for l-tyrosine and PTYR coatings. For this reason, a 4.0 mg/mL concentration of l-phenylalanine and 2-phenylethylamine was used to produce PFA and PEA coatings. Other reagents were used in proportional concentrations as described previously for PTYR. Independent optimization of the process parameters for these coatings was not applied.

**Figure 3:**
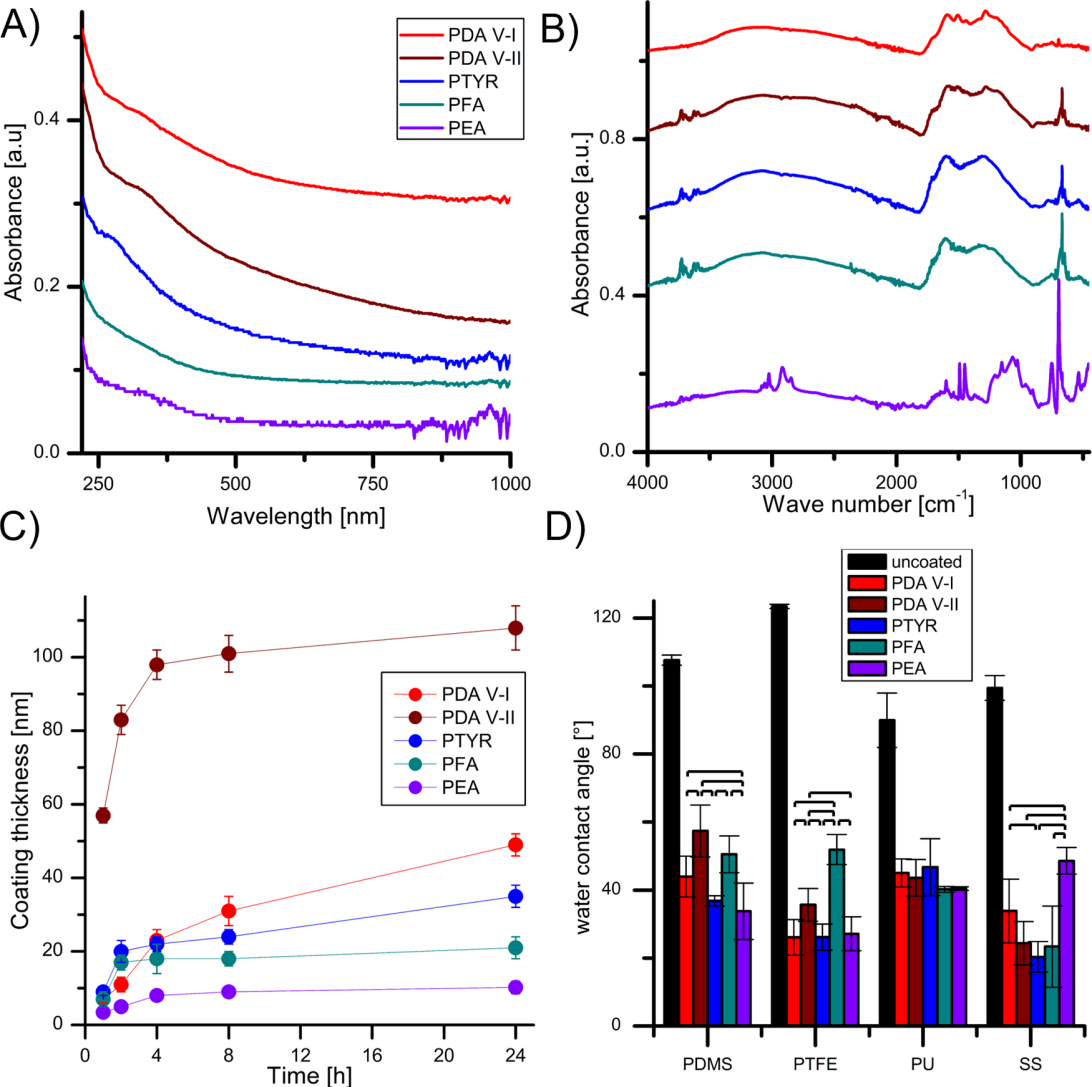
Comparison of the properties of polycatecholamine coatings. (A) UV-Vis spectrum of polycatecholamine films formed on the inner walls of quartz spectrophotometric cuvettes; (B) FTIR-ATR spectrum of polycatecholamine powders; (C) the AFM thickness measurement of the polycatecholamine coatings on the silicon surface in the function of coating formation time; (D) water contact angles of various materials coated with polycatecholamines. The legend for charts (A) and (B) is the same. Brackets in chart (D) indicate values statistically different (*p* < 0.05).

The proposed new polycatecholamines, analogous to PDA, form brown-black coatings on a wide variety of materials, as shown in Figure S2. The comparison of the properties of polycatecholamine coatings is presented in Figure 3 and Table 1. The UV-Vis spectra of all polycatecholamine coatings have a very similar shape (Figure 3A), showing a very weak pick around 300 nm for PDA coatings and around 270 nm for the rest of the coatings tested. According to the literature, pick at 270 nm can be addressed to the phenol state, while around 300 nm to the phenoxy form.^19^ The application of FTIR analysis (4000–400 1/cm) to the final products can be regarded as direct evidence of polymerization occurrence. The FTIR analysis is shown in Figure 3B, where the red color represents dopamine polymerized by atmospheric air (PDA V-I), and the wine color represents dopamine polymerized by the addition of sodium periodate NaIO_4_ (PDA V-II). Blue, cyan, and violet represent products of Fenton polymerization of l-tyrosine, l-phenylalanine, and 2-phenylethylamine, respectively. The FTIR analysis of two PDA products (PDA V-I and PDA V-II) shows no difference in the most critical bands (hydroxyl region around 3600 1/cm, and amine regions 3500–2800 1/cm and 1800–900 1/cm^20^), which confirms the uniform formation of the polymer matrices in both cases. The FTIR analysis of subsequent phenylamines (l-tyrosine, l-phenylalanine, 2-phenylethylamine) shows a broad pattern similar to PDA, which confirms the polymerization occurrence. The absorption shifts in the aromatic, aliphatic, and amine regions are driven by the differences in the chemical structure (i.e., the presence of the carboxylic group in l-tyrosine and l-phenylalanine around 1600 1/cm) of used phenylamines. Nonetheless, the presence of C=O and C–O stretching absorption bands highlights the oxidation step of selected amines, which is attributed as a critical step for their polymerization, which can be supported by the presence of a VU-Vis peak at about 300 nm (Figure 3B). The signals in the region of 3700 1/cm are addressed to the amplified noise of the detector.

**Table 1:** The Roughness of Polycatecholamine Coatings

	PDA V-I	PDA V-II	PTYR	PFA	PEA
Ra [nm]	50 ± 10^a^	23 ± 1^a^	12 ± 1^b^	12 ± 4^b^	7 ± 2^b^
R_Z_ [nm]	369 ± 64^a^	195 ± 18^a^	133 ± 26^b^	101 ± 27^b^	84 ± 10^b^

a Values statistically different (*p* < 0.05) from each other in the group, including PDA V-I and PDA V-II.

b Values statistically not different (*p* > 0.05) from each other in the group, including PTYR, PFA, and PEA.

The thickness of the polycatecholamine coatings as a function of the process time is significantly different for various coatings, as shown in Figure 3C. Representative AFM images of the edges of each tested coating created by removing a portion of the coating with a steel razor blade and plots of the difference in height between the coated and uncoated areas are shown in Figure S3. The thickest coating was obtained with periodate-oxidized dopamine (PDA-VII), where 108 ± 6 nm of the coating thickness was formed after 24 h of the process. According to this method, other authors have shown similar results, obtaining thicknesses in the range of 90–110 nm after 24 h of the process.^8,21^ The atmospheric oxygenated dopamine (PDA V-I) gives a coating of about 50 nm in thickness after 24 h, which is also consistent with the literature.^1,11,21^ A similar coating thickness (57 ± 2 nm) can be obtained after only 1 h of the periodate-oxidized dopamine polymerization with our selected reaction conditions (PDA V-II), which makes this process much more efficient.

Atmospheric oxidation of dopamine (PDA V-I) is a relatively slow process where the thickness of the coating increases continuously along with reaction time due to the limited oxygen solubility in water. On the other hand, the chemical oxidation of phenylamines (PDA V-II, PTYR, PFA, and PEA) is a much faster process, owing to a not limited amount of oxidant in the solution by its dissolution. In this case, the coating thickness increases significantly only during the first 4 h of the process. We do not observe any increase in the thickness of the PFA and PEA coatings after this time, probably due to the total consumption of phenylamine substrates. However, an increase in PTYR coating thickness between 4 and 24 h of the polymerization process is present: 22 ± 2 and 35 ± 3 nm, respectively. For this phenylamine, there is a limitation in the substrate availability for the oxidation reaction due to the limited solubility of l-tyrosine in water. We use a supersaturated l-tyrosine solution, so the oxidized part of the substrate is replaced by a fresh portion dissolved from the suspension. The PTYR coating is produced longer, but the highest increase in coating thickness is still in the first 4 h of the process. The significant difference in the thickness of PDA V-II and polycatecholamines produced by chemical oxidation (PTYR, PFA, and PEA) is probably due to differences in the polymerization mechanism. l-Tyrosine polymerization requires one more oxidation step than dopamine to introduce a second hydroxyl group into the carbon ring. l-Phenylalanine and 2-phenylamine require the oxidative introduction of two hydroxyl groups into the ring before polymerization (see Mechanism paragraph).

As shown in Figure 3D, each polycatecholamine coating causes a significant hydrophilization of the surface of PDMS, PTFE, PU, and SS. The water contact angle value was reduced by almost 80% for the most hydrophobic PTFE and about 50% for the least hydrophobic PU. In the case of PU, all polycatecholamine coatings hydrophilize the surface similarly (*p* > 0.05). For all other materials, there are significant differences (*p* < 0.05) in the water contact angles between the individual coatings. The difference in the interaction between the coating and the material’s surface becomes more noticeable when the material is more hydrophobic.

**Figure 4:**
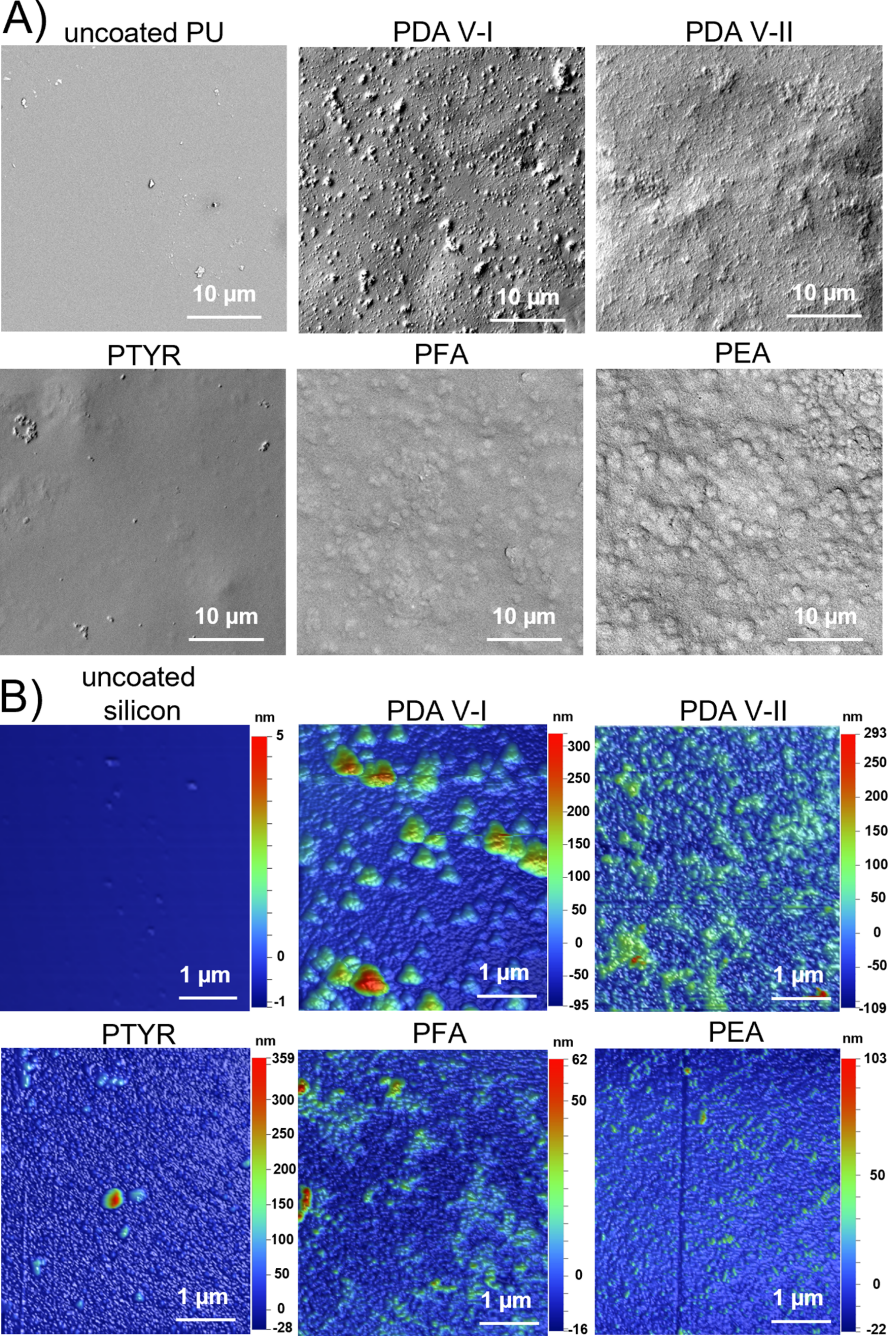
SEM (A) and AFM (B) images of polycatecholamine coatings surface topography. The coatings were synthesized on PU and silicon for SEM and AFM observations, respectively.

The PDA coating produced by the atmospheric oxidation of dopamine (PDA V-I) is characterized by the highest roughness, as shown in Table 1. The values of the Ra and R_Z_ parameters of this coating are significantly higher (*p* < 0.05) than those of periodate-oxidized dopamine (PDA-VII). High roughness results from the presence of PDA nanoparticles permanently embedded on the surface of the coating. We have observed this phenomenon before in the case of PDA coatings on PU fibrous materials, with a higher number of nanoparticles for PDA V-I than for PDA V-II coatings.^22^ Lower values of roughness parameters were measured for PTYR, PFA, and PEA coatings made by Fenton oxidation. The mean values of both roughness parameters do not show a statistically significant difference (*p* > 0.05) for these three coatings. It seems that the rate and type of phenylamine oxidation influence the roughness of the coating. The slowest atmospheric oxidation produces the roughest coating, while the fastest Fenton oxidation produces the least rough coatings, regardless of the substrate used. This is confirmed by the SEM (Figure 4A) and AFM (Figure 4B) surface topography observations, where a higher roughness of the PDA (V-I and V-II) coatings surface can be observed than that for PTYR, PFA, and PEA coatings. We identified a significantly lower number of nanoparticles deposited on the coatings produced by the Fenton reaction. Representative AFM images used for coatings roughness parameter determination are shown in Figure S3.

**Figure 5:**
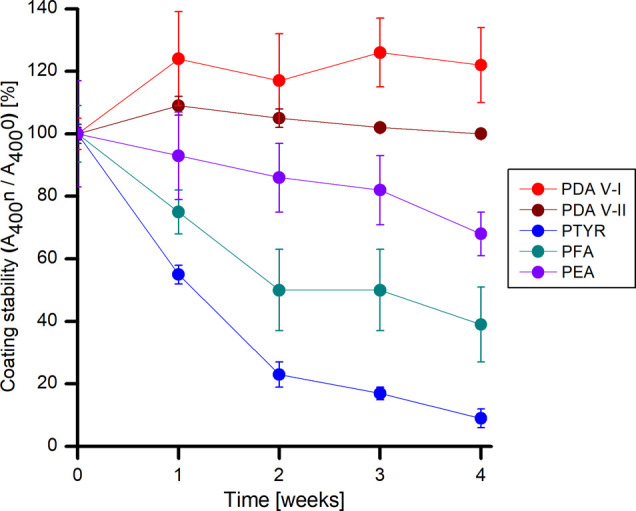
Stability of polycatecholamine coatings at pH 7.4 PBS at 37 °C. *A*_400_*n* and *A*_400_0—the absorbance at 400 nm of the polycatecholamine coatings at the tested time point and before the start of the experiment, respectively.

One of the essential properties of all types of coatings is their stability. Figure 5 shows the stability of polycatecholamine coatings during their incubation in 0.01 M PBS solution (pH 7.4) at 37 °C. Both PDA coatings show excellent stability under these conditions, regardless of the method of dopamine oxidation (PDA V-I and PDA V-II). PEA coating is less stable, showing 68% of the initial absorbance after 4 weeks. The PFA and PTYR coatings show the lowest stability, with 39% and 9% of initial absorbance after 4 weeks, respectively. As mentioned earlier, the initial adhesion of the oligomers formed in the first steps of the oxidation and polymerization of phenylamines is responsible for the adhesion strength of the entire coating to the surface. The adhesion of these oligomers must depend on the chemical structure of the starting phenylamines. Based on stability studies, we assumed that the weakest adhesion is achieved for oligomers resulting from the oxidation of substrates containing carboxyl groups (PTYR and PFA). This observation should be the subject of future research.

**Figure 6:**
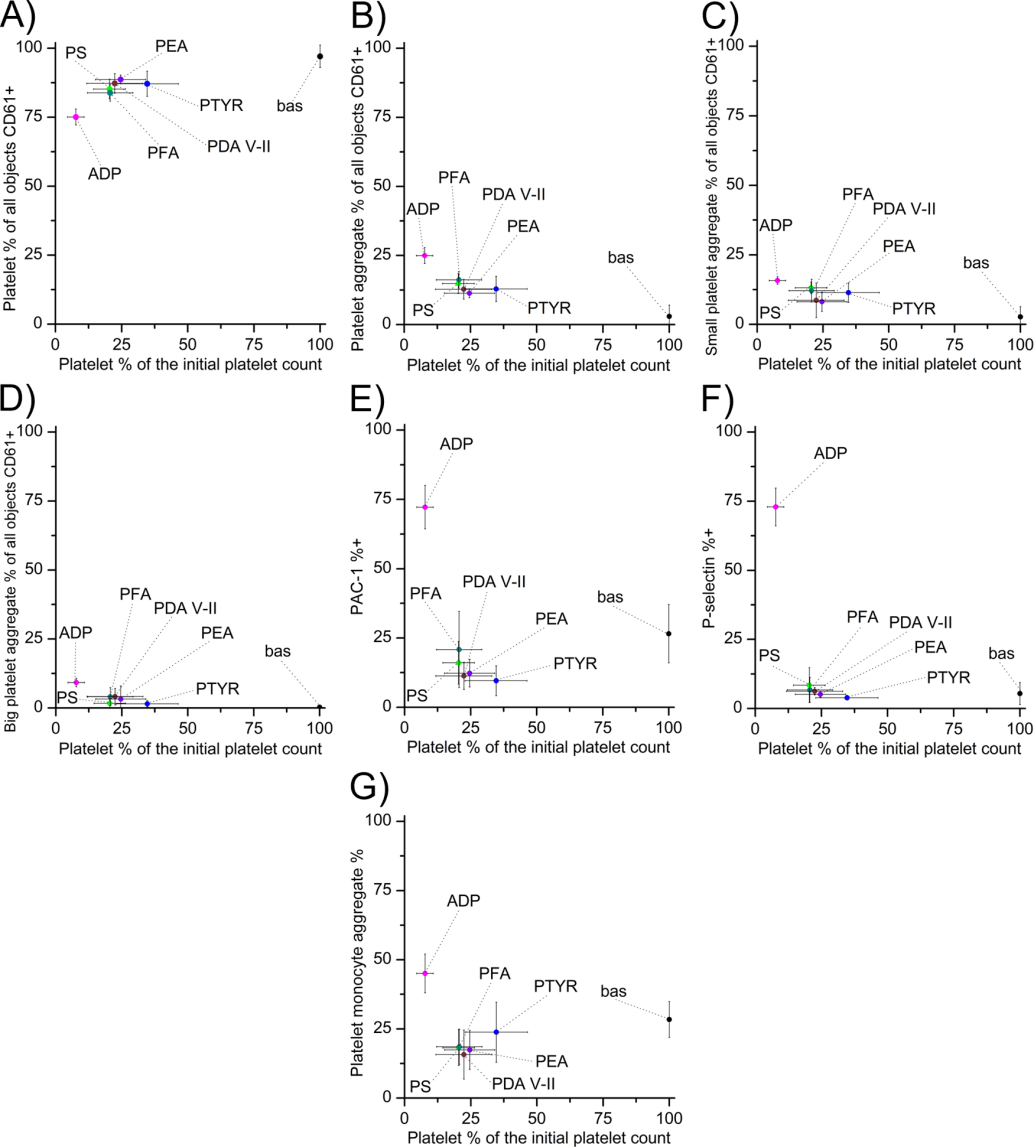
Hemocompatibility evaluation of polycatecholamine coatings. (A) Percentage of platelets of all CD61-positive objects; (B) platelet aggregate percentage of all CD61-positive objects; (C) small platelet aggregates of all CD61-positive objects; (D) big platelet aggregates of all CD61-positive objects; (E) expression of the active conformation of glycoprotein IIb/IIIa (PAC-1); (F) expression of P-selectin; (G) platelet-monocyte aggregates (%).

The tested polycatecholamine coatings were subjected to hemocompatibility evaluation. Blood–material interaction was evaluated as the percentage of blood platelets CD61-positive remaining in the blood after the shear test (Figure 6A). The analyses consider platelet–platelet aggregates (Figure 6B), which also distinguish small platelet aggregates, i.e., two plates forming an aggregate (Figure 6C), and big platelet aggregates, i.e., more than two platelets (Figure 6D). Platelet activation plays a vital role in diseases of thromboembolic origin. During activation, various surface glycoproteins are expressed on platelets. These include P-selectin, one of the most important markers of platelet activation. It is a transmembrane α-smooth muscle protein that is translocated to the platelet surface following activation. The activation of the tested materials was measured based on the expression of the active conformation of glycoprotein IIb/IIIa (PAC-1) (Figure 6E) and P-selectin (Figure 6F). Analysis of P-selectin activation shows that most coatings had little effect on activation processes without using remaining platelets in the blood. The second IIb/IIIa receptor showed similar activation properties. Activated platelets release many vasoactive and prothrombotic factors from intraplatelet granules.

Assessing the likelihood of creating monocyte-platelet aggregates is even more informative than the probability of creating microparticles. The interaction between leukocytes and platelets occurs only through the interaction of endothelial P-selectin and leukocyte PSG-1 glycoprotein. Specifically, monocytes are involved in the formation of leukocyte-platelet aggregates, which suggests that monocyte-platelet aggregation is a more sensitive test for platelet activation than P-selectin surface expression. Degranulation causes rapid loss of P-selectin. Moreover, the creation and detachment of the so-called microparticles plate (micro-plate) is important since these fragments possess antigens, CD61 and CD41, capable of forming a functionally efficient GPIIb/IIIa fibrinogen receptor. Platelet monocyte aggregation as a function of the percentage of platelets is presented in Figure 6G.

All tested polycatecholamines show good hemocompatibility properties, measured under hydrodynamic conditions. The results are in the region of the negative control material (bas—unactivated blood tested under static conditions; blood sample not activated by the introduction of shear forces). The best hemocompatibility results were obtained with PTYR. However, a minor concern is the sizeable statistical error indicating the need to refine reproducible coating properties, especially for blood morphotic elements.

**Figure 7:**
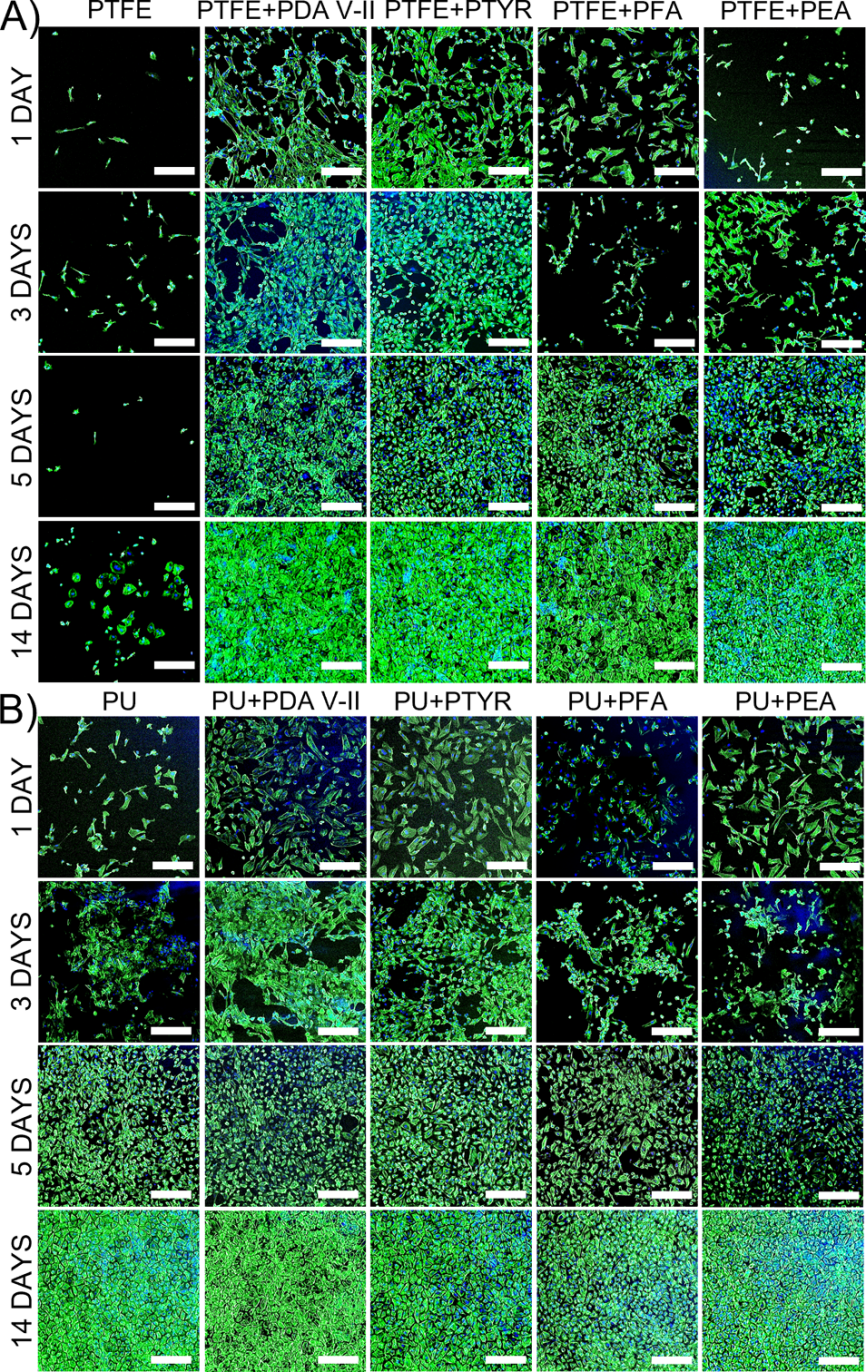
Confocal laser scanning microscope images of HUVECs growth on the surface of PTFE (A) and PU (B) uncoated and coated with polycatecholamines after 1, 3, 5, and 14 days of cells culture. The scale bar represents 200 μm.

Presented polycatecholamines were also analyzed for their ability to promote cell adhesion and proliferation. HUVECs were cultured on the surface of PTFE and PU coated with tested polycatecholamines. The cell’s morphology and proliferation rate were analyzed after 1, 3, 5, and 14 days using confocal laser scanning microscopy. As shown in Figure 7A, there is a significant difference in the cell adhesion capacity depending on the surface modification in the case of PTFE. After 1 day of cell culture, the number of cells is significantly higher on coated materials compared to unmodified PTFE. They also better adhered to the surface, especially in the cases of PTFE coated with PDA, PTYR, and PFA. On PEA-coated PTFE, cells were smaller than on other tested modifications but still bigger than on unmodified PTFE, where the round shape of the cells demonstrated a limited interaction and adhesion to the material. After 3 days post-seeding, the smallest number of cells was still observed on unmodified PTFE. Cell culture developed best on materials coated with PDA and PTYR, as indicated by the number and quality of cell adherence to the surface visible in the images. The cells were well spread and colonized the majority of the surface. The same results on the 3 days of cell culture were obtained before for PDA-coated and unmodified PTFE vascular scaffolds.^23^ In the first 3 days of cell culture, the number of adhered cells on the surface of PTFE coated with PFA and PEA is significantly lower than on PDA and PTYR coatings. As we showed, PFA and PEA coatings are much thinner than PDA and PTYR and are significantly less stable than PDA coatings. It is highly probable that PFA and PEA coatings degrade, exposing areas of raw material before cells can adhere. PTYR coating is also much less stable than PDA but thicker than PFA and PEA coatings. This probably allows the cells to adhere to the PTYR coating before it degrades enough to expose the raw material. After 5 days, the number of cells and their morphology were very similar on each modified material, covering almost the entire surface of the tested materials. On unmodified PTFE, the number of cells dropped compared to day 3. After 14 days, cells growing on PTFE coated with polycatecholamines reached full confluence and were tightly attached to the surface. There was a significant increase in the number of cells on unmodified PTFE, and their morphology changed. The cells became bigger and better attached to the material, but still not as much as on modified materials. After analyzing the confocal images, it is apparent that cells had partially detached and rolled up on unmodified PTFE. Some cells could be washed during the fixation and staining, as they were not permanently attached to the surface. This observation is similar to results obtained by Mi et al.^24^ They observed 8.4 times more of the HUVECs on the surface of PDA-coated PTFE in comparison to uncoated PTFE on day 14 of cell culture. The cell adhesion and proliferation on the surfaces modified by PTYR, PFA, or PEA have not been analyzed before. The obtained results indicate that these polycatecholamines can be an attractive alternative to PDA in the modification of materials in tissue engineering.

In comparison to PTFE, there was no such significant difference in the number and morphology of the HUVECs on the PU surface whether the material was modified (Figure 7B). After 1 and 3 days, cells had similar morphology and adhered well to the surface regardless of the material. However, there were noticeably more cells on material coated with PDA and PTYR than on unmodified PU. After 3 days of culture, cell morphology on PU coated with PEA and PFA has changed. Cells had a more spherical shape. This may be the consequence of degradation of the PFA and PEA coatings and exposing areas of raw material as previously described for cell culture on PTFE. In other variants, this phenomenon was much less visible. After 5 days, the cells covered almost the entire surface, regardless of the tested material, reaching full confluence after 14 days. Tsai et al.^25^ cultured chondrocytes on PDA-modified PU and obtained a 5% increase in the number of cells after 1 day versus uncoated material, which corresponds to our results. However, after 12 days of culture, this difference increased to 23%. In our study, such a difference could not be observed due to full confluence even on unmodified PU, which is mainly related to a different type of cultured cells. The lack of significant differences between unmodified and modified PU may result from the fact that the PU foil itself shows high biocompatibility and is hydrophilic, which promotes cell adhesion to such surfaces.^26^ For this material, the presence of additional polycatecholamine coatings does not bring significant benefits.

## 4 Mechanism

**Scheme 1:**
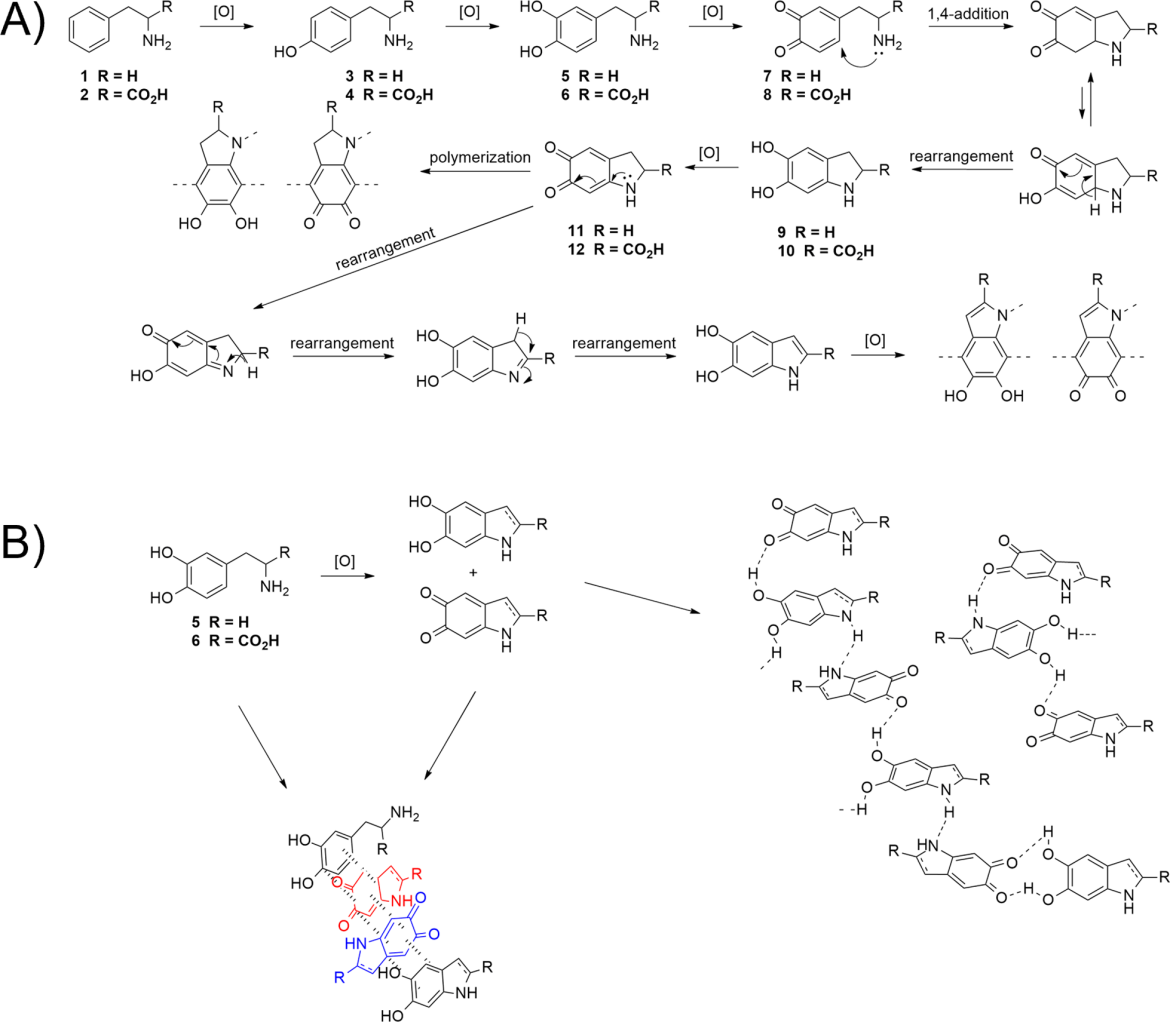
(A) Proposed Mechanism of Covalent Polymerization of Phenylamines; (B) Proposed Non-Covalent Interactions Between Monomers in Oxidative Polymerization of Phenylamines

The phenomenon of oxidative polymerization of dopamine (Scheme 1A-5) is well known, and the chemistry of this process has been tried to explain in numerous articles.^1,7,27−33^ Despite the widespread use of PDA, the mechanism of its formation is still a contentious issue due to the complex morphology and chemical composition of PDA itself. It has been well studied that oxidative polymerization of dopamine is a pH-dependent reaction that undergoes easily in mild basic conditions in the presence of oxygen. As described in the previous sections, other oxidants can support this process even in acid conditions. Nevertheless, all of them lead to PDA through multiple reaction steps. The first and essential one is the oxidation of dopamine to dopamine quinone (Scheme 1A-7, R = H) and intramolecular Michael-type addition, which gives leucoaminochrome (Scheme 1A-9) and dopaminochrome (Scheme 1A-11). These molecules, supported by further oxidation and rearrangement reactions, lead to polymeric forms of dopamine where heterocyclic units are bonded covalently.^18,34−37^ Alternative structural models have been proposed based on the ability of the monomers to bond in a non-covalent manner.^29,38,39^ Authors of this concept suggest that PDA consists of heterocyclic monomers and creates polymeric supramolecular aggregates that are held together by a combination of charge transfer, hydrogen bonds, and π stacking interactions (Scheme 1B). Based on the research of Hong et al.,^29^ it seems reasonable to assume that PDA formation can result from both self-assembly of monomers (dopamine and its oxidized forms) and oxidative covalent polymerization. However, further analysis of the PDA structure and formation kinetics of its components should be further investigated for scientific clarification.

Complementary to mentioned methods of dopamine polymerization, an enzyme-catalyzed reaction has to be added, which is a matter of great importance because of the formation of eumelanin in living organisms based on the catalytic oxidation of l-tyrosine by tyrosinase.^40,41^ Based on the biochemical example of eumelanin synthesis, in our case, the initial step of oxidative l-tyrosine polymerization is probably the hydroxylation of the aromatic ring at position C3, which leads to obtaining levodopa (Scheme 1A-6, R = CO_2_H). This compound contains a catechol-like aromatic structure and allows for oxidative polymerization, similar to dopamine. Our assumption seems fair because the Fenton reagent is known for hydroxylation of aromatic rings.^42,43^ An essential work of Raper proved the presence of the corresponding dihydroxy components regarding oxidation of l-tyrosine, tyramine, and l-phenylalanine using the Fenton reaction.^44^l-Tyrosine oxidation by hydrogen peroxide in the presence of iron(II) chloride resulted in obtaining a dark-brown suspension. After a laborious workup, the product of hydroxylation at position C3 in the aromatic ring, i.e., levodopa, was obtained, which was proven by extracting and analyzing this product from the reaction mixture. There are also other examples of metal-catalyzed oxidation of l-tyrosine, l-phenylalanine, and 2-phenylethylamine that rationalize the hypothesis of mono- and dihydroxylation as the first initial step of the proposed polymerization.^45−49^ All of these suggest, in this condition, that the hydroxylation of the aromatic ring occurs, and then the oxidative polymerization of levodopa may occur with a similar mechanism to dopamine (Scheme 1, R = CO_2_H). It is likely that both l-phenylalanine and 2-phenylethylamine may undergo a similar mechanism. This hypothesis, however, needs further studies and experimental investigation toward mechanism clarification.

## 5 Conclusions

The most common method of PDA coating synthesis is the oxidation of dopamine solution in an alkaline environment using atmospheric oxygen as an oxidizing agent. Application of other oxidation schemes (periodate, Fenton reaction) in acidic conditions allows to conduct this process significantly faster and to employ different phenylamines. We showed that the atmospheric oxygenated dopamine at pH 8.5 (PDA V-I) gives a coating of about 50 nm in thickness after 24 h, while a similar coating thickness can be obtained after only 1 h of our optimized periodate-oxidized dopamine polymerization in acidic conditions (PDA V-II).

l-Tyrosine, l-phenylalanine, and 2-phenylethylamine can be polymerized to form coatings on many materials, making them hydrophilic and biocompatible. The coatings have been effectively produced on the surface of PDMS, PTFE, PU, stainless steel, glass, and silicon, proving the versatility of their application. The new polycatecholamine coatings show similar properties to the well-known ones obtained from PDA. The coating process results in significant hydrophilization of the material’s surface for each polycatecholamine coating used. The adhesion strength of the coatings to the material depends on the chemical structure of the phenylamine used, which results in different coating stabilities. The stability is the highest when using phenylamines without carboxyl groups. However, carboxyl-containing coatings (PTYR, PFA) can be beneficial for further chemical surface modification and attachment of biologically active molecules. The coating’s surface roughness is also significantly different between polycatecholamines. The high number of nanoparticles deposited on the surface of PDA coatings makes them rough on the nanoscale, which can be beneficial in tissue engineering applications. PTYR, PFA, and PEA coatings are smoother, making them potentially more suitable for blood-contacting medical devices. All tested polycatecholamines show good hemocompatibility properties, measured under hydrodynamic conditions. Based on the results obtained, the degree of influence of the tested material on the initiation of blood activation and aggregation processes is low. Cell cultures showed that all tested polycatecholamines promote HUVEC adhesion and proliferation. This effect is especially visible for materials with a surface unfavorable to cell growth, like PTFE. The coating of PTFE with polycatecholamines caused full confluence on the surface after 5 days of culture, whereas for the uncoated material, only single cells were visible even after 14 days. The best results were achieved for PDA and PTYR, where the surface was almost completely covered with cells after just 3 days of cell culture. The cell layer is continuous, without defects, after 14 days of culture for both tested materials (PTFE and PU). This means that even less stable coatings made of phenylamines containing carboxyl groups (PTYR, PFA) are suitable for modifying materials used in tissue engineering. The rapid adhesion and proliferation of cells on these coatings probably prevent the polycatecholamines from being washed away from the surface of the coated material. All experiments show that the new polycatecholamine coatings have great potential in many biomedical applications, especially in blood-contacting devices or tissue scaffolds, and are an economically beneficial alternative to the widely used PDA. Our discovery that l-tyrosine, l-phenylalanine, and 2-phenylethylamine can be much cheaper components for multifunctional coating and can replace dopamine in usage on a bigger scale not only starts a wide range of opportunities for obtaining functional materials but also can be another scientific challenge regarding the mechanism and structural investigation. The synthesis of PDA-analogous from various phenylamines makes it possible to create coatings containing many functional groups, depending on the chemical structure of the substrate used. This opens up excellent possibilities for the biomaterial’s surface functionalization to give them the desired properties, regardless of the type of the modified surface.

## Supporting Information Available

Influence of the used oxidizing agent on the rate of PDA film formation; the results of experiments for the selection of PTYR synthesis parameters according to the Box–Behnken plan; pictures of various materials with polycatecholamine coatings; representative AFM images of the silicon surface coated with PDA V-I (A), PDA V-II (B), PTYR (C), PFA (D), and PEA (E); images of the edge created by removing a portion of the coating with a steel razor blade, plots of the difference in height between the coated and uncoated areas, and images of the coatings topography analysis (PDF)

## Abbreviations

ADP: adenosine diphosphate
FBS: fetal bovine serum
HUVECs: human umbilical vein endothelial cells
PBS: phosphate-buffered saline
PDA: polydopamine
PDMS: polydimethylsiloxane
PEA: polyphenylethylamine
PFA: polyphenylalanine
PMMA: polymethyl methacrylate
PS: polystyrene
PTFE: polytetrafluoroethylene
PTYR: polytyrosine
PU: polyurethane
SDS: sodium dodecyl sulfate
SS: stainless steel
