# Supplementary material for: Polymerization of l-Tyrosine, l-Phenylalanine, and 2-Phenylethylamine as a Versatile Method of Surface Modification for Implantable Medical Devices
Source: ACS Omega. 2022 Oct 20;7(43):39234–49. doi: 10.1021/acsomega.2c05289 (PMC9631876; doi:10.1021/acsomega.2c05289)
Supplement: Supplementary file 1 — ao2c05289_si_001.pdf [file ao2c05289_si_001.pdf]

Supporting Information

Polymerization of ʟ-Tyrosine, ʟ-Phenylalanine, and 2-Phenylethylamine as a Versatile Method of Surface Modification for Implantable Medical Devices

Kamil Kopeć[a,*], Agata Ryżko[a,b], Roman Major[c], Hanna Plutecka[d], Justyna Więcek[c], Grzegorz Pikus[e], Jakub W. Trzciński[a,f] Adrianna Kalinowska[a], and Tomasz Ciach[a]

[a] Faculty of Chemical and Process Engineering, Biomedical Engineering Laboratory, Warsaw University of Technology, Waryńskiego 1, Warsaw, Poland

[b] Department of Cytology, Faculty of Biology, University of Warsaw, Miecznikowa 1, Warsaw, Poland

[c] Institute of Metallurgy and Materials Science, Polish Academy of Sciences, Reymonta 25, Cracow, Poland

[d] Department of Medicine, Jagiellonian University Medical College, Skawińska Str. 8, Cracow, Poland.

[e] School of Chemistry, University of Bristol, Cantock's Cl, Bristol BS8 1TS The United Kingdom

[f] Centre for Advanced Materials and Technologies CEZAMAT, Warsaw University of Technology, Poleczki 19, Warsaw, Poland

*Correspondence: Kamil Kopeć: kamil.kopec@pw.edu.pl.

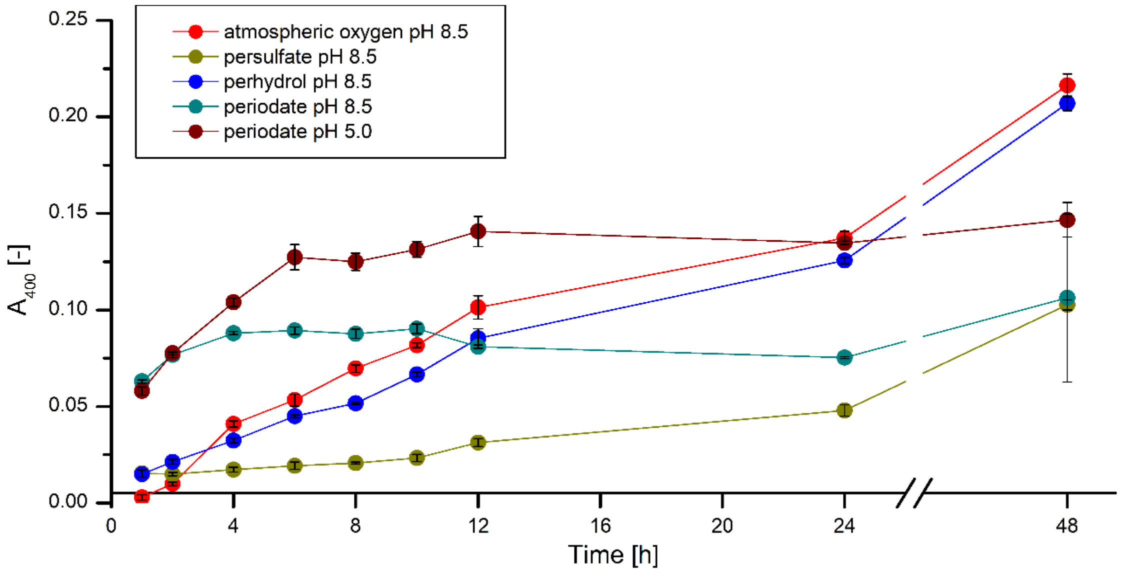

Figure S1. Influence of the used oxidizing agent on the rate of PDA film formation.

Table S1. The results of experiments for selecting PTYR synthesis parameters according to the Box-Behnken plan. The absorbance at 400 nm was measured after 1 h of the PTYR coating synthesis process on the inner walls of the PMMA spectrophotometric cuvette.

Experiment number	pH [-]	$FeCl_2$ concentration [mM]	H_2O_2 : $FeCl_2$ molar ratio [-]	A_{400} [-]
1	2	0.1	25	0.001
2	6	0.1	25	0.008
3	2	0.9	25	0.012
4	6	0.9	25	0.000
5	2	0.5	5	0.005
6	6	0.5	5	0.014
7	2	0.5	45	0.004
8	6	0.5	45	0.016
9	4	0.1	5	0.004
10	4	0.9	5	0.031
11	4	0.1	45	0.004
12	4	0.9	45	0.027
13	4	0.5	25	0.034
14	4	0.5	25	0.034
15	4	0.5	25	0.033

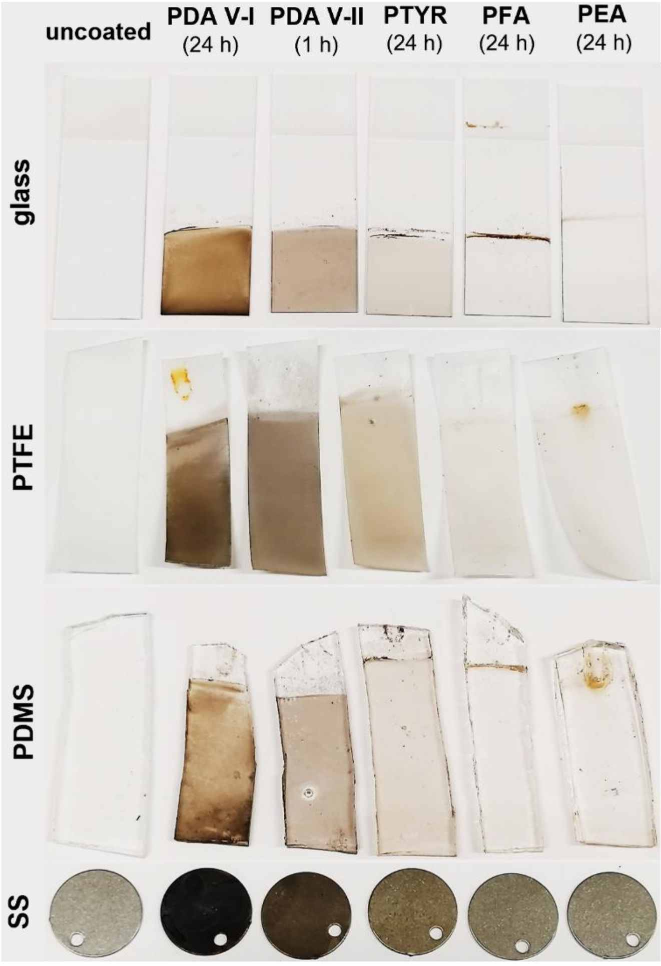

Figure S2. Pictures of various materials with polycatecholamine coatings.

A)

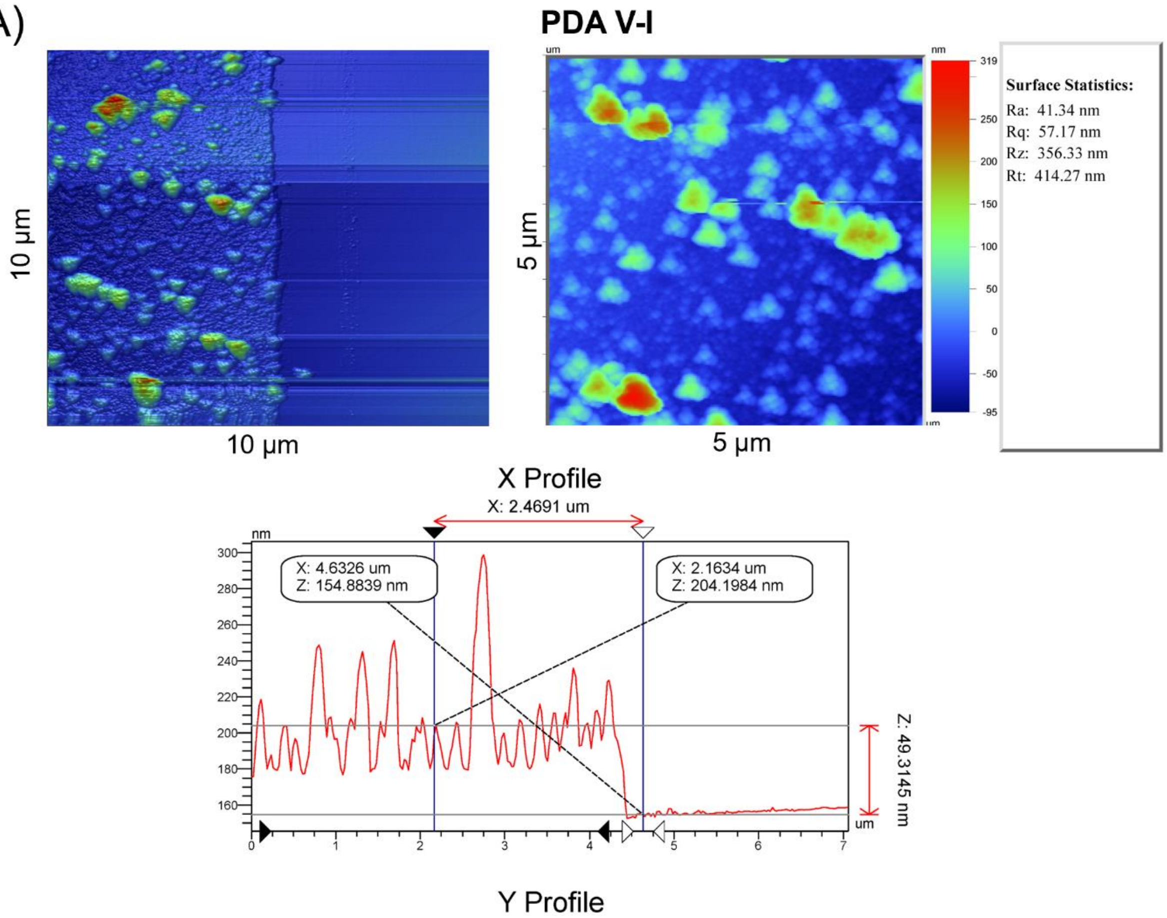
PDA V-I
10 µm
10 µm
5 µm
5 µm
Surface Statistics:
Ra: 41.34 nm
Rq: 57.17 nm
Rz: 356.33 nm
Rt: 414.27 nm
X Profile
X: 2.4691 um
X: 4.6326 um
Z: 154.8839 nm
X: 2.1634 um
Z: 204.1984 nm
Z: 49.3145 nm
Y Profile

B)

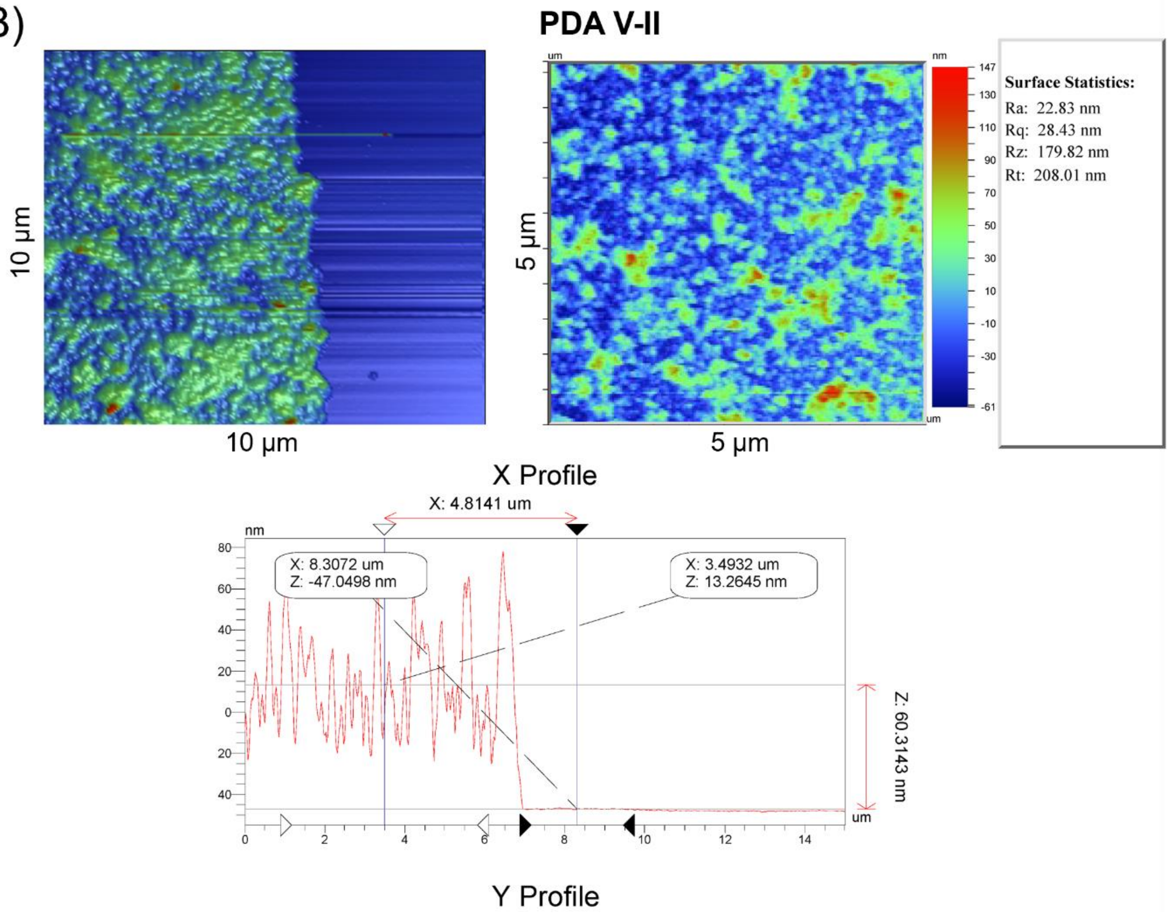
PDA V-II
10 µm
10 µm
5 µm
5 µm
Surface Statistics:
Ra: 22.83 nm
Rq: 28.43 nm
Rz: 179.82 nm
Rt: 208.01 nm
X Profile
X: 4.8141 um
X: 8.3072 um
Z: -47.0498 nm
X: 3.4932 um
Z: 13.2645 nm
Z: 60.3143 nm
Y Profile

C)

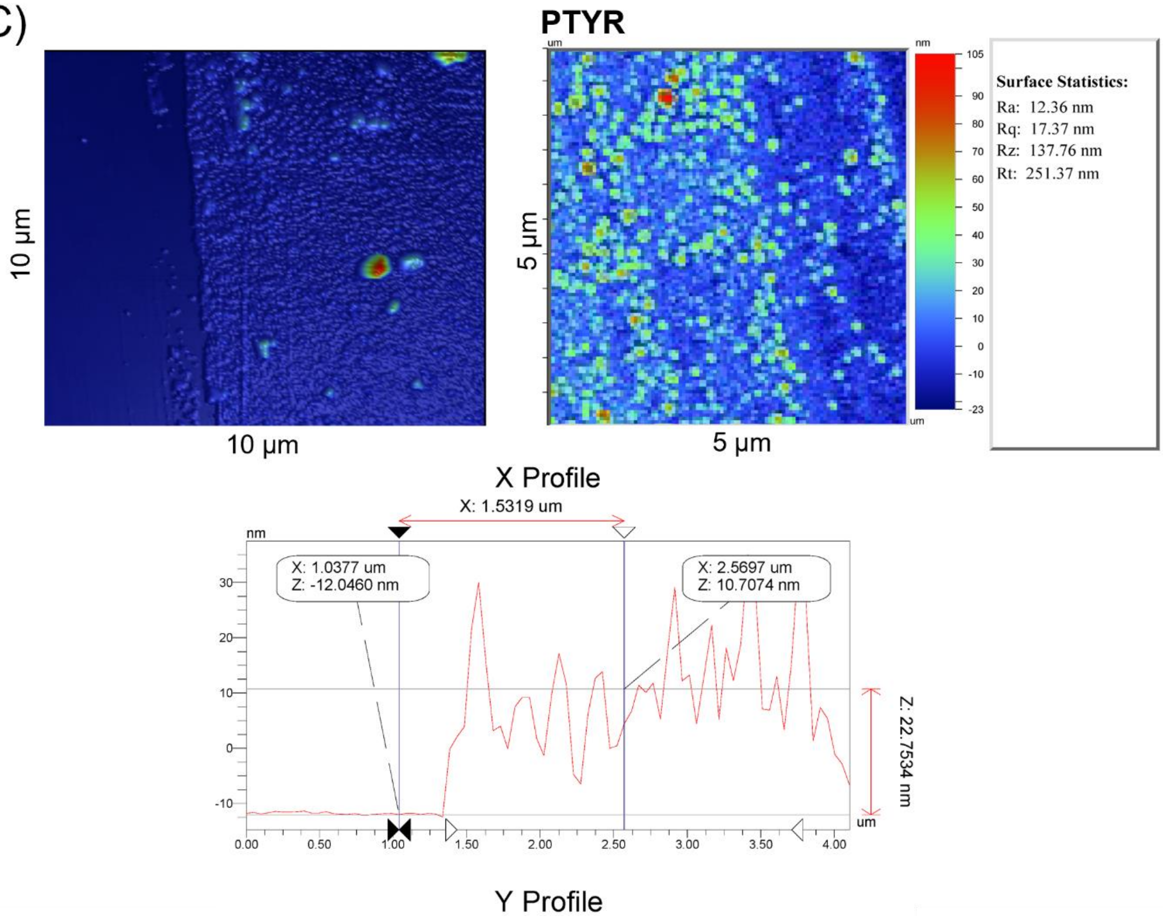
PTYR
10 µm
10 µm
5 µm
5 µm
Surface Statistics:
Ra: 12.36 nm
Rq: 17.37 nm
Rz: 137.76 nm
Rt: 251.37 nm
X Profile
X: 1.5319 um
X: 1.0377 um
Z: -12.0460 nm
X: 2.5697 um
Z: 10.7074 nm
Z: 22.7534 nm
Y Profile

D)

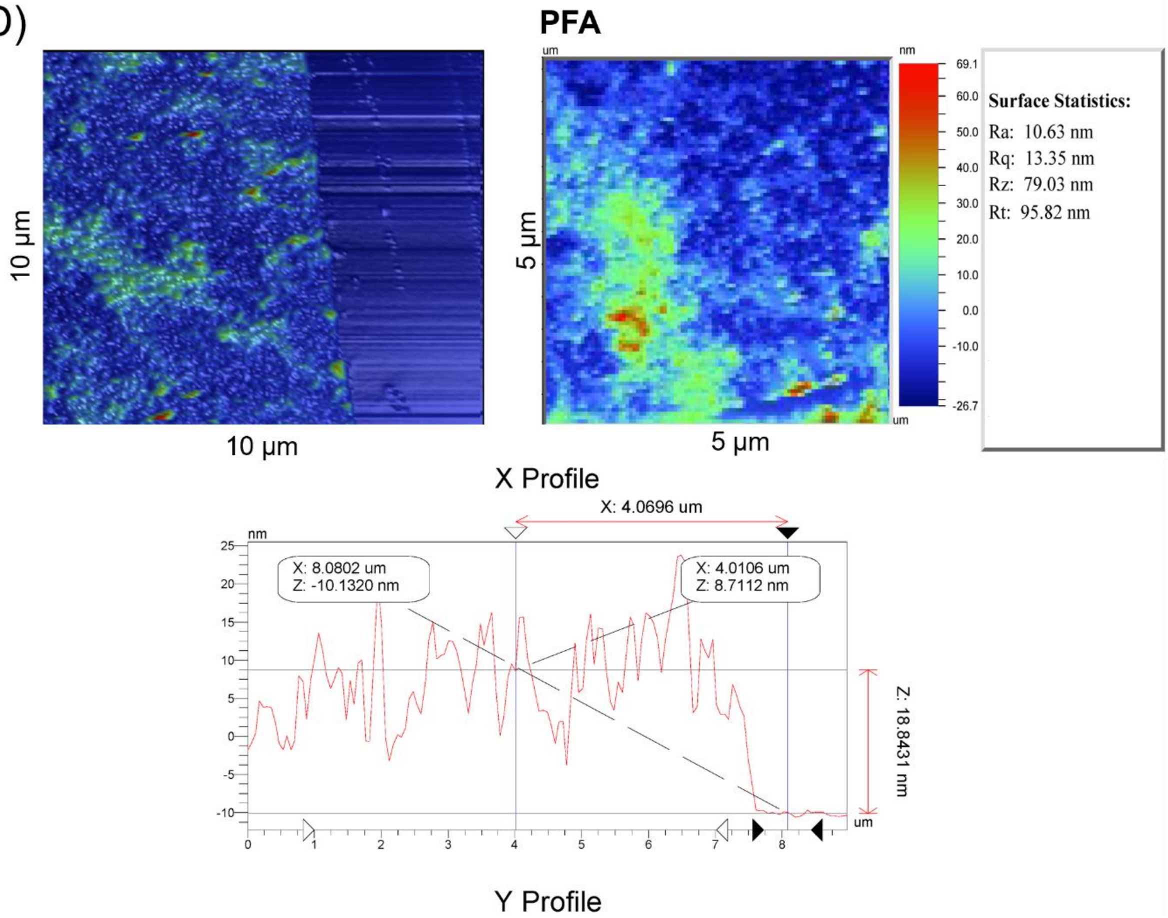
PFA
10 µm
10 µm
5 µm
5 µm
Surface Statistics:
Ra: 10.63 nm
Rq: 13.35 nm
Rz: 79.03 nm
Rt: 95.82 nm
X Profile
X: 4.0696 um
X: 8.0802 um
Z: -10.1320 nm
X: 4.0106 um
Z: 8.7112 nm
Z: 18.8431 nm
Y Profile

E)

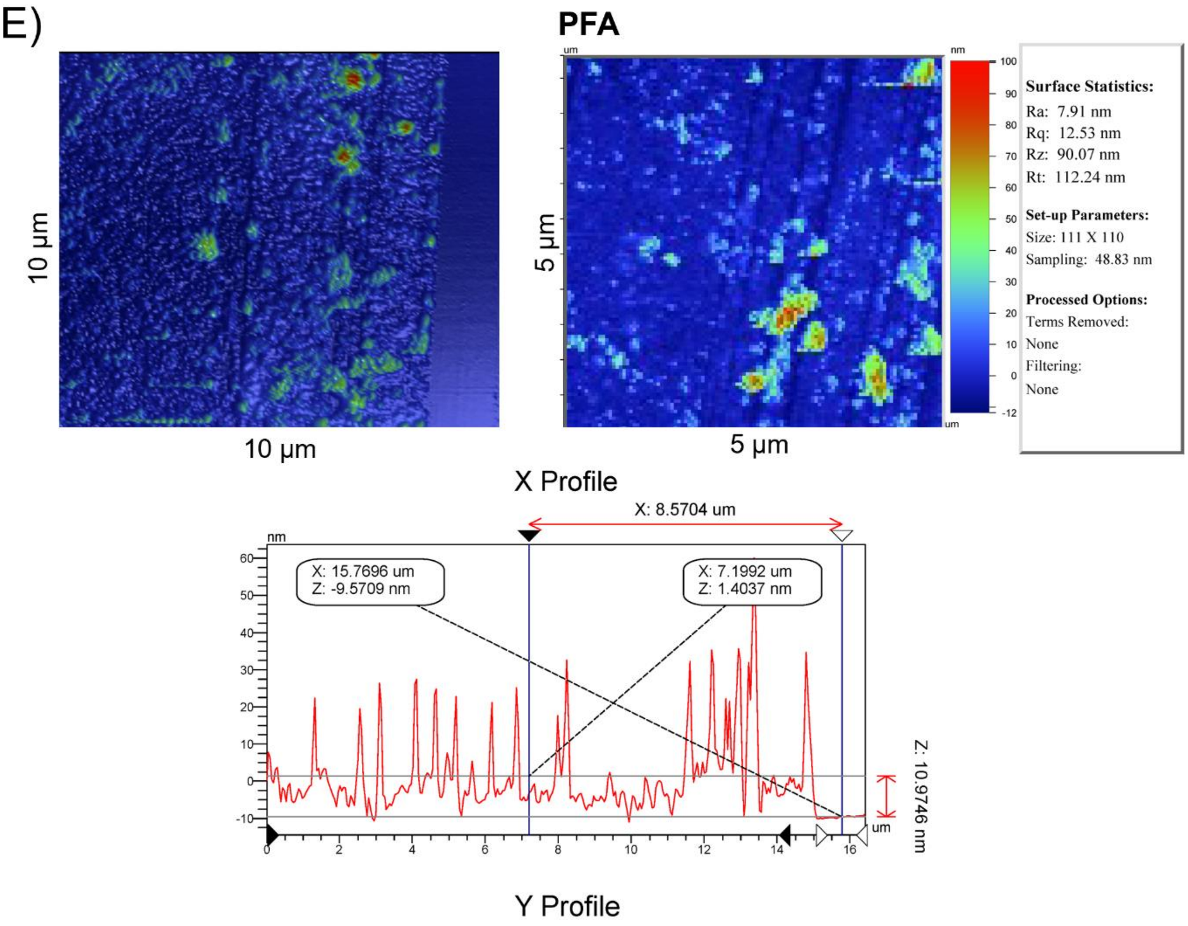

Figure S3. Representative AFM images of the silicon surface coated with PDA V-I (A), PDA V-II (B), PTYR (C), PFA (D), and PEA (E). Images of the edge created by removing a portion of the coating with a steel razor blade, plots of the difference in height between the coated and uncoated areas, and images of the coatings topography analysis. One of five measurements for each coating is presented.
